# The Transactions—The Association, Etc.

**Published:** 1894-11

**Authors:** 


THE TRANSACTIONS, THE ASSOCIATION, ETC.—The position the JOURNAL occupies in relation to the State Association is peculiar. It is somewhat embarrassing from the fact that the senior editor was for some years the Secretary, and although he resigned in the hope of promoting greater harmony in the ranks, was re-elected and again resigned—thus making all suspicion of "sour grapes" out of the question—there are those ready to attribute to envy, spite or prejudice, any criticism of his successor's work that the JOURNAL may make, however fair, and with whatever *assurances* of best intentions, and a consideration alone of the Association's best interest. Why should we envy Dr. West the position? It is at best a hard and almost a thankless one,—or being earnestly desirous of advancement, why should we want to hinder the Secretary in the furtherance of his ideas of progress? Though when the progress seems to us backward we just can't help speaking out.

We hesitated about writing the criticism, fearing some such construction; but, as all old readers know, the JOURNAL has, for years devoted much of its space to association matters, and has worked, in season and out of season, for its advancement, and to fail now to criticise what the fairest and most indifferent member must charcterize as an error of judgment, whereby the members are put to still further and unnecessary expense, would be inconsistent with the JOURNAL'S record and its avowed course and policy. It has been the Association's champion and defender, and in the language of the day, we have "made a specialty" of looking to what we conceived to be its best interests. To say nothing would perhaps be the wiser course, and the one we feel much inclined in future to follow; it is not our funeral.

In this issue we publish a statement from the President with reference to an editorial item in our last. It will readily be understood. We publish it with pleasure, and again disclaim all intention to reflect upon the Secretary or of doing him injustice. What was said in the editorial referred to was the result of the impression left on our mind after a brief conversation with Dr. McLaughlin. In reply to our inquiry why the Secretary did not publish his picture, he said that Dr. West had informed him that it would add to the cost, and being already overdrawn, it was thought best not to publish it; words about to that effect. In substance we were right; the only difference being the President authorized the omission, which lifts the responsibility from the Secretary's shoulders. A distinction without a difference.

DR. COLEMAN has a short article on organization in this issue. It is indirectly a reply to the JOURNAL'S several editorials on the subject. Let it be here understood, the JOURNAL has not counseled abandoning the State Association. We have submitted a suggestion that perhaps, in view of the fact that at the end of thirteen (twenty-three?) years of trial, and of earnest endeavor the Association is but a trifle larger in members than in 1882 (1870), it would be best to give it up, and stimulate local organization, with the view of having once a year a big medical convention of delegates, somewhat after the style of political conventions. The JOURNAL suggested that in event of such convention the cost for membership would be obviated, and that a dollar *ante* from each delegate would pay expense of hall, etc.; we have not advised reducing the membership fee, as Dr. Coleman seems to infer. If any one knows or can suggest a better plan the JOURNAL will gladly second it. Or, if the members prefer to let well enough alone (is it "well enough" when, for some reason the membership is being rapidly diminished?) we have nothing to say in opposition; but with a membership of 354* the Association can not pay for many more thousand dollar editions of its Transactions, nor longer hold together. Can some plan be suggested whereby members can be *induced* to attend? Our observation has been that as a rule only those who attend, pay dues; hence a large contingent is annually dropped from the rolls. Let the interested members follow Dr. Coleman's example and give the JOURNAL their views on "how can the profession of Texas be organized into a State Association."

---

### Medical News and Miscellany.

**Dr. T. N. Skeen** has removed from Wichita Falls to Winsboro, Texas.

* Dr. Coleman makes an error both in the number of members, putting it at 386, and the total number of physicians in Texas; the best estimates put the latter at plus 5000. That, of course, lowers the per cent.

